# Brain extraction from cerebral MRI volume using a hybrid level set based active contour neighborhood model

**DOI:** 10.1186/1475-925X-12-31

**Published:** 2013-04-12

**Authors:** Shaofeng Jiang, Weirui Zhang, Yu Wang, Zhen Chen

**Affiliations:** 1 Key Laboratory of Nondestructive Testing (Nanchang Hangkong University), Ministry of Education, NanChang Hangkong University, NanChang 330063, P.R China

**Keywords:** Brain extraction, Hybrid level set, Active contour neighborhood, MRI volume

## Abstract

**Background:**

The extraction of brain tissue from cerebral MRI volume is an important pre-procedure for neuroimage analyses. The authors have developed an accurate and robust brain extraction method using a hybrid level set based active contour neighborhood model.

**Methods:**

The method uses a nonlinear speed function in the hybrid level set model to eliminate boundary leakage. When using the new hybrid level set model an active contour neighborhood model is applied iteratively in the neighborhood of brain boundary. A slice by slice contour initial method is proposed to obtain the neighborhood of the brain boundary. The method was applied to the internet brain MRI data provided by the Internet Brain Segmentation Repository (IBSR).

**Results:**

In testing, a mean Dice similarity coefficient of 0.95±0.02 and a mean Hausdorff distance of 12.4±4.5 were obtained when performing our method across the IBSR data set (18 × 1.5 mm scans). The results obtained using our method were very similar to those produced using manual segmentation and achieved the smallest mean Hausdorff distance on the IBSR data.

**Conclusions:**

An automatic method of brain extraction from cerebral MRI volume was achieved and produced competitively accurate results.

## Background

Brain extraction or skull stripping is an important pre-procedure in many neuroimaging analyses. Any subsequent analysis, such as registration between fMRI and T1-Weighted MRI, measurement of brain volume, brain tissue classification into White Matter, Gray Matter and GSF, and cortical surface reconstruction, will be highly dependent on the robustness and precision of the brain masks which are generated in the brain extraction step. Manual brain extraction requires an operator with professional knowledge about cerebral anatomy, and is extremely time-consuming. As a consequence, many automatic and semi-automatic brain extraction techniques using image processing have appeared in recent years. These require the operator to provide a few initial parameters prior to running the extraction process. These techniques can generally be categorized into three types: region-based, boundary-based and hybrid methods. We briefly describe and compare these methods as follows.

The region-based methods divide the cerebral image into several regions using thresholding or clustering techniques by observing that the intensities of the voxels belonging to the same tissue are similar. The brain region can be extracted from these regions by morphological operations and region merging. Lemieux [1] proposed an algorithm which utilizes several intensity thresholds and morphological operations to remove the non-brain areas. Coxs [2] used a Gaussian mixture model to estimate an intensity range in order to extract the brain areas in a slice-by-slice manner. Hahn and Peitgen [3] presented a pre-flooding height based watershed algorithm to group image voxels with similar intensities then merged the largest connected components to obtain the brain volume. The region-based methods are usually sensitive to image processing parameters and image artifacts, such as noise and intensity inhomogeneity. Therefore, the region-based methods usually require the user to determine the proper initial parameters.

The boundary-based methods are used to locate a closed contour which partitions the cerebral image into the internal part (brain) and the external part (non-brain). The contour can be a tessellated mesh or a continuous curve. Smith [4] proposed a well-known boundary-based method called the Brain Extraction Tool (BET). BET initializes the brain contour with a tessellated mesh and pushes the mesh to the brain boundary with some smoothing forces and a pushing force. BET is relatively insensitive to parameter settings because of the globally defined pushing force. The main drawback of BET is that the brain boundary is often over-smoothed due to the global pushing force. Another widely used boundary-based method is Brain Surface Extraction (BSE) [5]. BSE uses a series of procedures to separate brain and non-brain tissues: anisotropic diffusion filtering, Marr-Hildreth edge detector and morphological operators. When using morphological operators the initial parameters can badly affect the accuracy of the extraction results. Many boundary-based methods use an active contour model which has resulted in great progress in medical image segmentation during recent years. Zhuang [6] developed an active contour model based level set method (MLS) that defines the image data function according to the pushing force used in BET and the smoothing function according to the mean curvature of the evolving curve. MLS can achieve good results in most cases. However, it is easy for MLS to leak through a weak boundary. Zhang [7] developed a brain extraction method using an improved region-based local binary fitting (LBF) model which is less sensitive to intensity inhomogeneity, but the extraction result can be over-smoothed by the leakage detection method when the rate of curvature is high.

The hybrid methods try to integrate region-based and boundary-based methods to improve the extraction result. Most of the hybrid methods are coarse to fine procedures. Usually the region-based method serves as a pre-process to obtain the rough brain region or boundary. The boundary-based method is carried out using the initial contour obtained in the pre-process. Extraction results using hybrid methods are more accurate since the initial contour is close to the brain boundary at the beginning of the boundary-based stage. Ségonne [8] proposed a hybrid watershed algorithm (HWA) combining a watershed algorithm and a deformable model, which applies the watershed algorithm to get an initial brain volume for use in the deformable model. The deformable model is driven by a smoothing force following the definition in BET. An atlas-based force is added to ensure that the deformed template possesses the shape of a brain within certain tolerances. Huang [9] applied an expectation-maximization algorithm to a mixture of Gaussian models to determine the initial brain contour for use in the geodesic active contour evolution. Rex [10] developed a brain extraction meta-algorithm (BEMA), which executes four extraction algorithms to obtain individual extraction results in concert with an atlas-based registration procedure, and combines the results to achieve the final improved brain extraction result using a variety of anatomically specified Boolean functions. It is obvious that BEMA is time consuming. To reduce the computational time required by atlas-based brain extraction, Eskildsen [11] proposed an atlas-based method using nonlocal segmentation techniques that eliminated the need for time-consuming non-rigid registrations. Sadananthan [12] used intensity thresholding to generate a preliminary binary mask ideally including the brain tissues, the skull and some thin connections between them. A graph cuts based image segmentation technique (GCUT) was then used to remove the narrow connections. GCUT is usually quite accurate but sometimes results in large errors due to following the wrong edge and the boundary of the brain is usually too smooth. Iglesias [13] proposed a robust, learning-based brain extraction system (ROBEX). ROBEX uses a random forest classifier trained to detect the brain boundary. The contour is then refined to obtain the final brain tissue results using graph cuts. ROBEX is quite robust, and major errors are rare (e.g., leaving the whole cerebellum out, or an eye in). The main disadvantage with ROBEX is that it produces over-smoothed results in brains with very convoluted surfaces on the gyri and sulci.

The above mentioned hybrid methods have achieved great improvements in the accuracy and robustness of automatic brain extraction techniques. However, none of these automatic or semi-automatic methods is a complete substitute for the manual method due to the appearances of over-smoothing, leakage through a weak boundary and missing brain tissue caused by local convergence. It is hard to solve these problems all at once. Brain extraction is a compromised problem where a semi-global understanding of the image is required as well as a local understanding [4]. For example, using local region features is effective in obtaining a sharp brain boundary and eliminating leakage, but can easily lead to local convergence at the edges between the white and gray matter. In order to try to overcome this conflict we proposed a new brain extraction method for T1-weighted MRI volumes.

## Methods

We developed a new, automatic method called the hybrid level set based active contour neighborhood model to extract brain tissue from T1-weighted MRI volume. Our method was inspired by the Graph Cuts Based Active Contours (GCBAC) proposed by Xu [14]. Xu defined the belt-shaped neighborhood region around a contour as the contour neighborhood (CN). This was obtained by slightly dilating the current contour. The closest contour that is a global minimum within its CN can then be found using graph cuts, giving an initial contour. The CN and the closest contour were iteratively replaced until the objective was achieved. In our work, we called the iteratively replaced CN an active CN (ACN). Our proposed method pre-processed T1-weighted MRI scans with BET to generate the initial contour and the ACN. Then, an improved hybrid level set model, using a nonlinear speed function which can eliminate the boundary leakage effectively, was defined in the ACN to obtain a new contour. Since the initial contour obtained by BET is close to the real brain boundary, the real brain boundary should be the global minimum within the ACN obtained from the initial contour. Thus, our proposed method should prove to be robust and accurate.

To extract the brain tissue from the 3D MRI volume an improved slice by slice solution was adapted to reduce time consumption and improve the extraction result. The solution used the resultant contour from the current slice to initialize the contour in adjacent slices by contracting or expanding the resultant contour of the current slice.

### Description of the ACN Model (ACNM)

**Figure 1:**
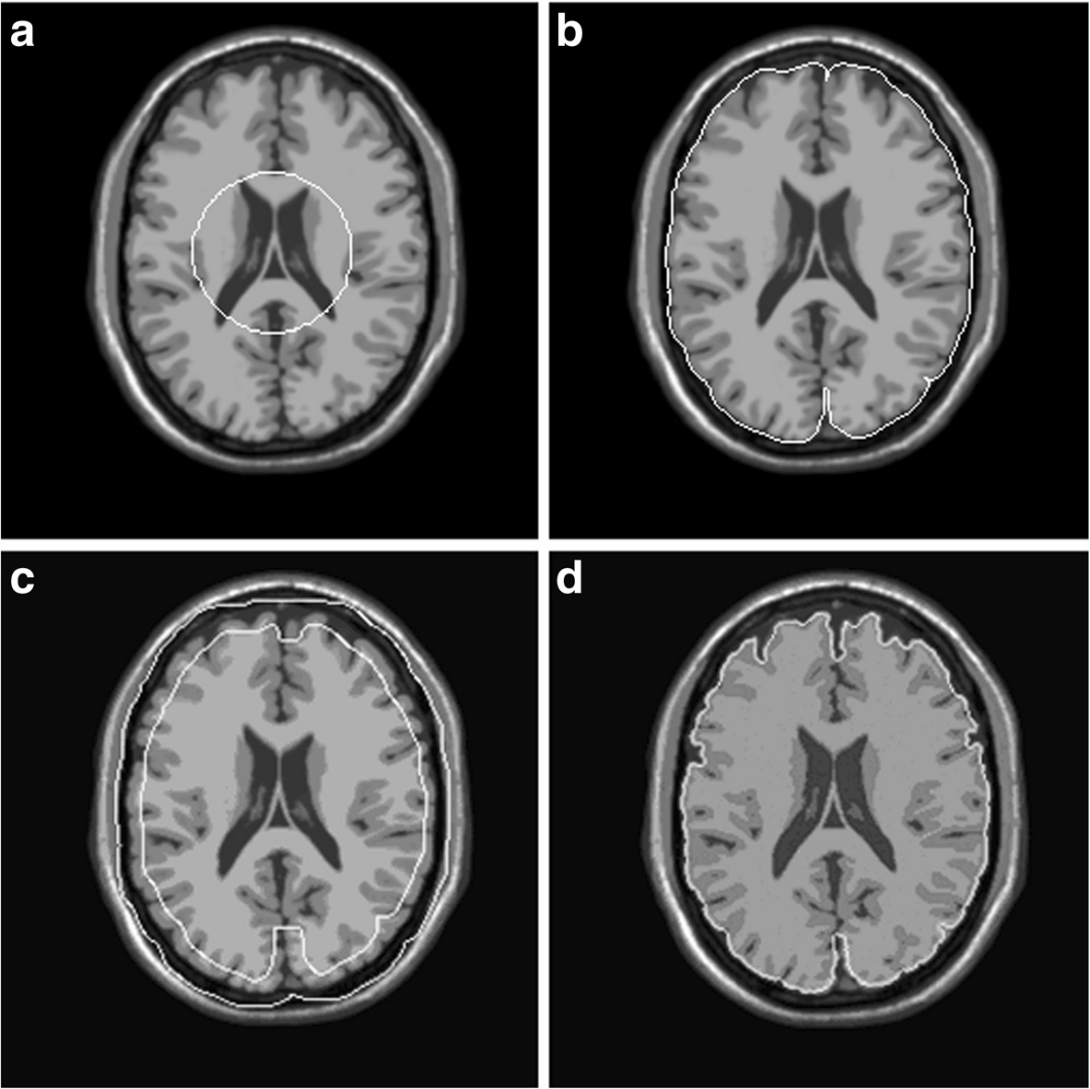
**Description of contour evolution in the ACN.** (**a**) the initial contour; (**b**) the brain boundary obtained by BET; (**c**) the ACN obtained by dilating the boundary in (**b**); (**d**) the brain boundary obtained by iteratively performing the hybrid level set model in the ACN.

The proposed method for 2D MRI images comprises the following major steps:

1. The initial contour was estimated using a modified BET method for 2D images [15]. The white contour in Figure 1(a) is the initial contour using BET. The white contour in Figure 1(b) is the brain boundary obtained using BET, which almost encloses the brain tissue, but is too rough to express the detail structure of the gyri and sulci. However, due to its robustness for obtaining the initial contour and parameters, BET still provides a good initial contour for brain extraction.

2. An initial ACN was defined by dilating the initial contour. In Figure 1(c), the region in the white ring is the initial ACN. Within the ACN, an improved hybrid level set was performed to obtain a new contour. The improved hybrid level set redefined the speed function of hybrid level set to avoid leakage through a weak boundary. Details of the improved hybrid level set will be described in the following sections.

3. The brain boundary was obtained by iteratively replacing the new contour and the ACN. In Figure 1(d), the brain boundary is more accurate than that in Figure 1(b) and better depicts the convoluted shape of the gyri and sulci.

### Initialization for 3D MRI volumes

**Figure 2:**
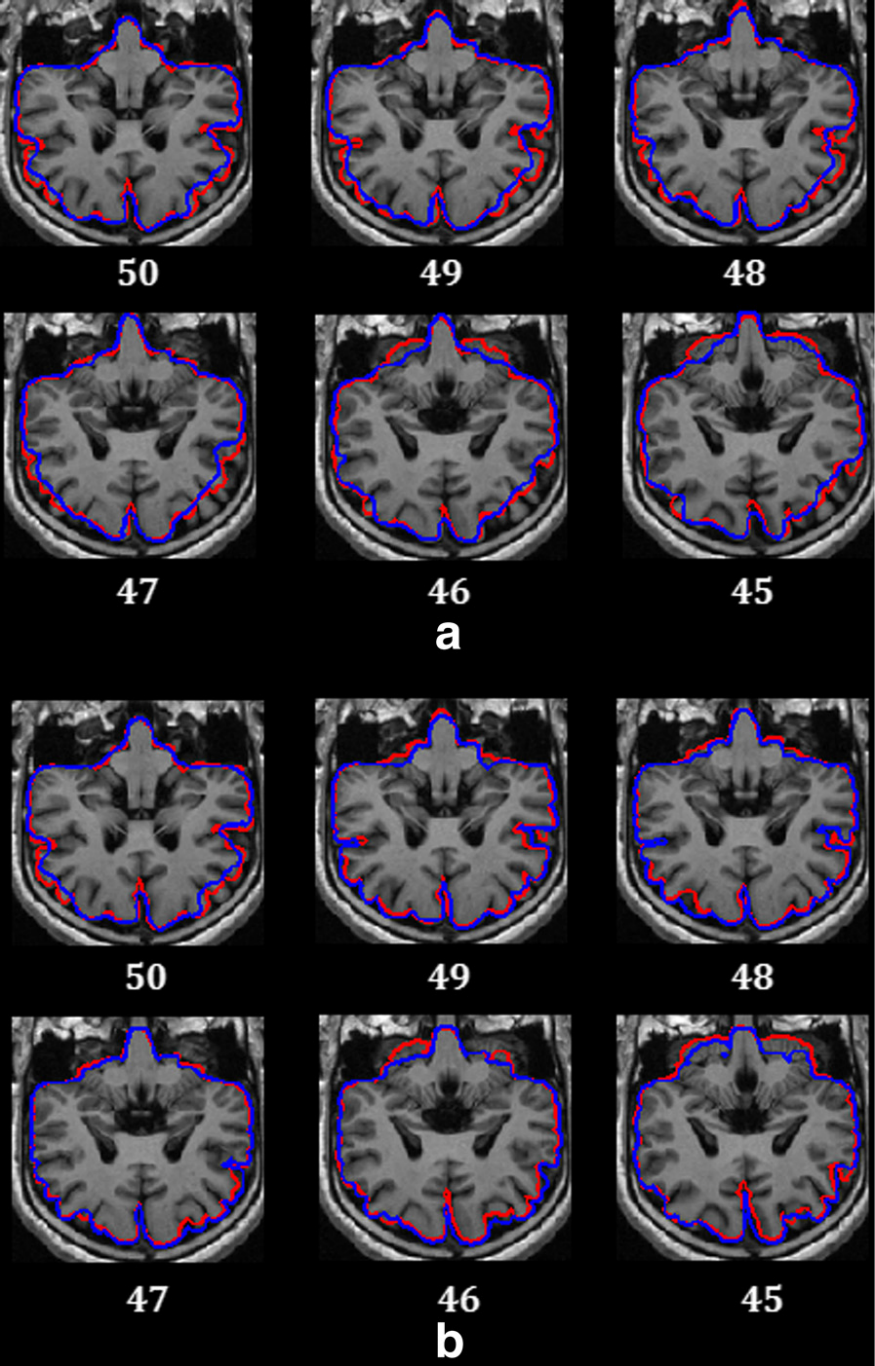
**Comparison of the slice by slice contour initial method with each contour initialized by BET.** (**a**) each contour initialized by BET. (**b**) the slice by slice brain contour initial method; Blue curve represents the initial contour and the red curve represents the resultant brain boundary.

For 3D MRI volumes, our method started with a slice in the middle of the volume. The initial contour for the middle slice was estimated using the modified BET for 2D brain images. For the residual slices, the initial contour was not estimated using the modified BET but using the resultant brain boundary of its adjacent slice with respect to the continuity of the brain surface. This is because the parameters in BET don’t fit all of the slices in the 3D volume and result in bad initial contours in slices containing the eyeball, brainstem and cerebellum part. In Figure 2(a), the initial contours for all of the slices were individually obtained using the modified BET hence all of the initial contours were badly initialized, leading to bad results in all of the slices. The initial method in our work is quite like the initial method used in [6]. However, the initial contour is always in the resultant brain boundary in [6]. Considering that in the 3D MRI volume, the brain boundary in the neighboring slice can be either inside or outside of the resultant boundary extracted from current slice, a new slice by slice initial method for 3D MRI volumes was proposed and this performed better than the initial method in [6]. In our work, the initial contour was obtained by contracting or expanding the current boundary using a simple discriminant method. The discriminant method first partitioned the image region where the intensity of the current slice is higher than that of the next slice, designating this R1 and the rest of the image region R2. Then, if the resultant brain boundary of the current slice covered R1 more than R2, the contour in the next slice was initialized using a contracted resultant brain boundary, otherwise, the resultant brain boundary was expanded.

**Figure 3:**
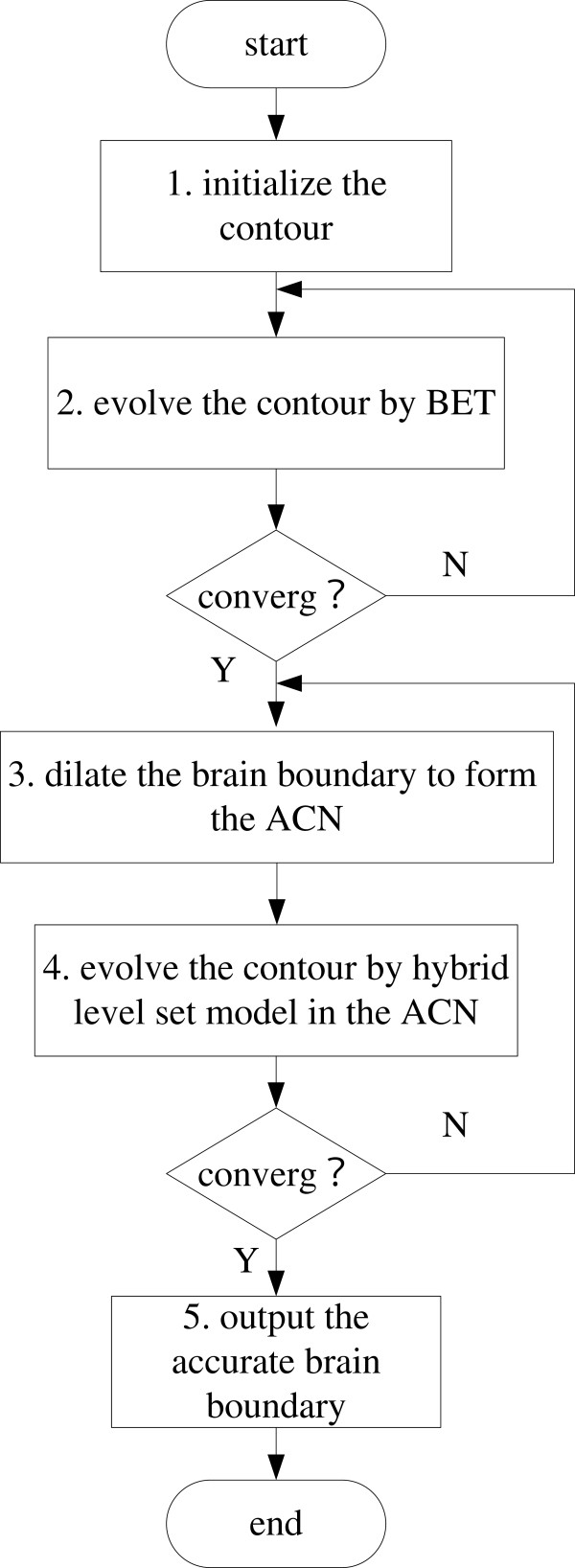
The flow chart for 2D brain extraction using ACNM.

**Figure 4:**
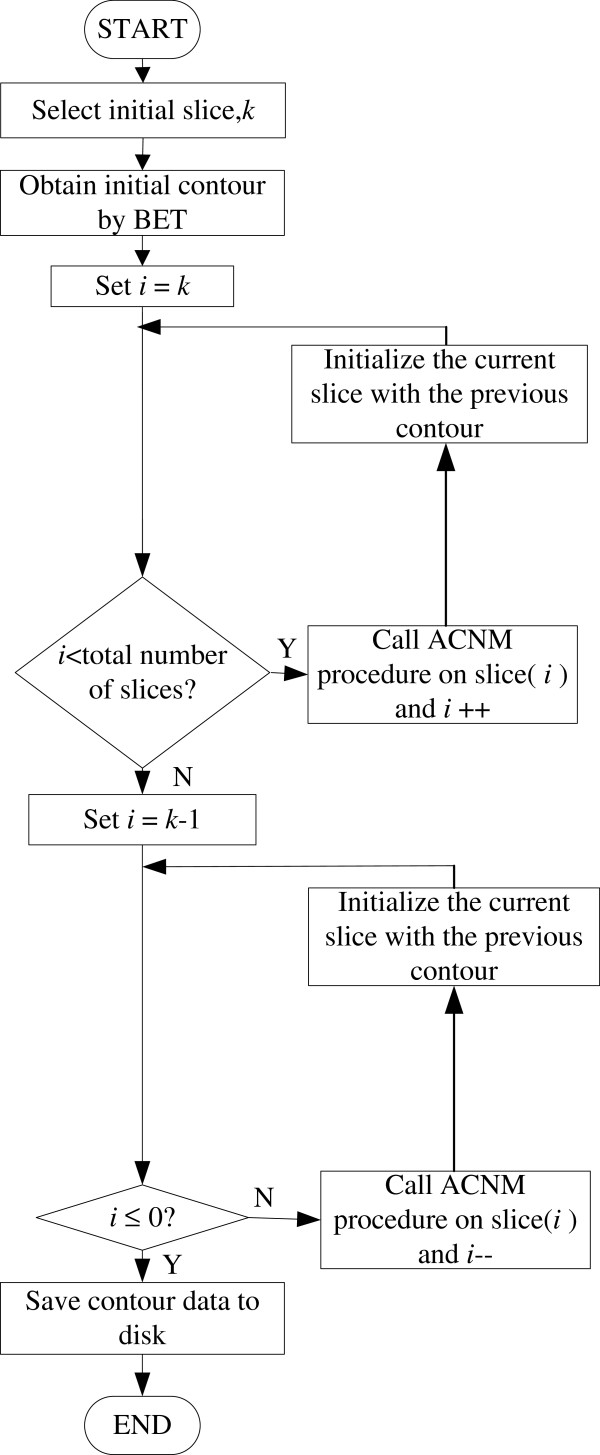
The flow chart for 3D brain extraction using ACNM.

The proposed initial method has some advantages. In the initial method, BET was only performed once on the middle slice. In most of MRI volumes, the brain tissues in middle slice are simpler than that found in other slices. The modified BET can immediately and robustly obtain the initial contour from the middle slice, but for other slices containing the eyeball, brainstem and cerebellum part a bad initial contour might be obtained. Setting the initial contour of the other slices from the resultant brain boundary of the previous slice can effectively trace the change of the brain boundary in the adjacent slices. Therefore, the initial contour is very close to the true brain boundary at the very beginning and this ensures that the active contour evolves to the true brain boundary using the hybrid level set model. This can save computational time and improve the accuracy of the results. In Figure 2(b), the initial contour in the 50th slice of the 3D MRI volume was obtained using the modified BET and the initial contours in the rest slices were well initialized using the resultant brain boundary from their adjacent slice. It can be seen that the brain boundaries in Figure 2(b) are more accurate than those in Figure 2(a). Figure 3 shows the flow chart for 2D brain extraction using ACNM. Figure 4 shows the flow chart for 3D brain extraction using ACNM.

### The hybrid level set model

The level set model has remarkable advantages for image segmentation. A lot of improved models have been developed in the level set framework for different image segmentation purposes and fields. Among these models, the boundary-based geodesic active contour model and region-based Chan-Vese model have made great improvements. Juan [16] introduced stochastic motion principles to minimize the object function in the Geodesic Active Contours framework in order to overcome the local minimum problem. Xie [17] proposed a Magnetostatic Active Contour Model, which defines a force based on magnetostatics and hypothesized magnetic interactions between the active contours and object boundaries. This model is able to capture complex geometries and multiple objects using a single initial contour. Wang [18] proposed a new approach called the “fluid vector flow” active contour model which has the ability to capture a large range and extract concave shapes. The hybrid level set model [19] integrated both boundary and region information. The traditional function of the hybrid level set model is written below:

(1) $$E\left(\varphi \right)=-\alpha \int_{\Omega }\left(I-\mu \right)H\left(\varphi \right)d\Omega +\beta \int_{\Omega }g\left|\nabla H\left(\varphi \right)\right|d\Omega$$

*H*(*φ*) being the Heaviside function defined as

(2) $$H\left(\varphi \right)=\left\{\begin{array}{c} 0,\;\mathrm{if}\;\varphi <0\\ 1,\;\mathrm{if}\;\varphi \geq 0 \end{array}\right)$$

where *I* is the image to be segmented. *g* = *g*(| ∇ *I*|) is the boundary feature map related to the image gradient. *φ* is the level set function whose zero level set defines the active contour. *­Ω* represents the image domain. *α* and *β* are predefined weights to balance the two terms on the right hand side of the equation. The first term on the right hand side of the function is regarded as the region term where *μ* is the parameter related to the lower bound of the gray-level of the target object. The first term encourages the contours to enclose the regions with gray-levels greater than *μ*. The second term on the right hand side of the function is the geodesic active contour function represented in the level set formulation. The role of this term is to encourage the contours to attach to the areas with high image gradients. If *φ* is a signed distance function (SDF), i.e. |∇*φ* | = 1, the associated curve evolution PDE in the level set formulation can be simplified to

(3) $$\varphi_{t}=\alpha \left(I-\mu \right)+\beta \mathrm{div}\left(g\nabla \varphi \right)$$

The above equation can be considered as a speed function. Generally, the speed function consists of a combination of two terms: the data term and the smoothing term. Accordingly, the region term in equation (1) is the data term and the geodesic active contour function is the smoothing term.

**Figure 5:**
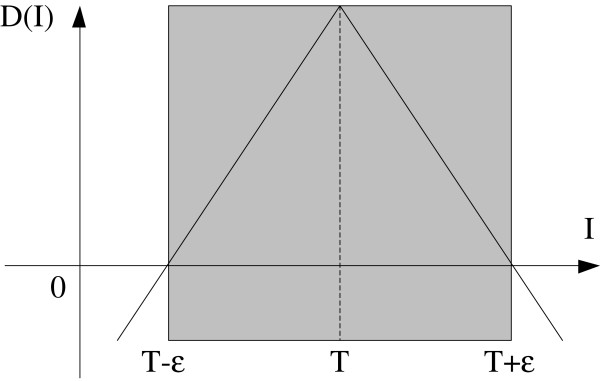
**A linear speed function based on image intensity.** It causes the model to expand over regions with grayscale values within the specified (positive) range and contract otherwise.

Lefohn [20] proposed a linear piecewise speed function, shown in equation (4), which depends solely on the grayscale value of the input data I at a point. T controls the brightness of the region to be segmented and *ε* controls the range of grayscale values around T that can be considered to be inside the object. In this way, a model situated on voxels with grayscale values in the interval *T* ± *ε* will expand to enclose these voxels, whereas a model situated on grayscale values outside that interval will contract to exclude these voxels. The speed term is gradual (refer to Figure 5) and thus the effects of D diminish as the model approaches the boundaries of regions with grayscale levels within the *T* ± *ε* range.

(4) $$\varphi_{t}=\left|\nabla \varphi \right|\;\left[\mathrm{\alpha D}\left(I\right)+\left(1-\alpha \right)\nabla \cdot \frac{\nabla \varphi }{\left|\nabla \varphi \right|}\right]$$

Where

$$D\left(I\right)=\varepsilon -\left|I-T\right|$$

### The improved hybrid level set model

The above mentioned hybrid level set model performs well for the segmentation of brain tissues. However, it has a few problems when applied to brain extraction. The first problem is that brain extraction is image processing where a semi-global understanding of the image is required as well as a local understanding. However, applying the hybrid level set model to brain extraction easily leads to local convergence. The second problem is that the weak boundaries between the brain tissues and surrounding tissues, with no efficient gradient, may make the gradient intensity based models [16-19] lose effectiveness, although some of these models have a robust convergence ability. The last problem is that the linear formulations for the data terms in equations (3) and (4) are too simple to describe the regional information and this often leads to leakage through a weak boundary.

To solve the last two problems a new nonlinear piecewise speed function, (shown in equation (5)), was defined to suit brain extraction in level set solvers. The data term *D*(*I*) in the new speed function depends on the grayscale value of input data I at a voxel, the mean grayscale value *μ* of the ACN, the global maximal value *I*_*m*_ in the ACN and three regional static values $I_{\max}$, *I*_*min*_ and *I*_*μ*_ in the neighborhood (*N*_*1*_ and *N*_*2*_) of the voxel. In our research the size of neighborhood *N*_*1*_ was 40 and the size of neighborhood *N*_*2*_ was 20.

(5) $$\varphi_{t}=D\left(I\right)+\beta \mathrm{div}\left(g\left(I_{r}\right)\nabla \varphi \right)$$

where

$$D\left(I\right)=\left\{\begin{array}{l} -\exp\left(-\frac{I-\mu }{I_{m}-\mu }t\right)\;\mathrm{if}\;I-\mu <0\\ \exp\left(-\frac{I-\mu }{I_{m}-\mu }t\right)-\exp\left(-\frac{2\left(I_{m}-\mu \right)-\left(I-\mu \right)}{I_{m}-\mu }t\right)\;\mathrm{if}\;I-\mu \geq 0\; \end{array}\right)$$

$$I_{r}=\sqrt{\frac{\left|I_{\max}-I_{\min}\right|+\left|I_{\mu }-I\right|}{2}}$$

$$\begin{array}{l} \mu =\mathrm{AVG}(I\left(\mathrm{ACN}\right))\\ I_{m}=\mathrm{MAX}(I\left(\mathrm{ACN}\right))\\ I_{\max}=\mathrm{MAX}(I\left(N_{1}\right))\\ I_{\min}=\mathrm{MIN}(I\left(N_{2}\right))\\ I_{\mu }=\mathrm{AVG}(I\left(N_{2}\right)) \end{array}$$

**Figure 6:**
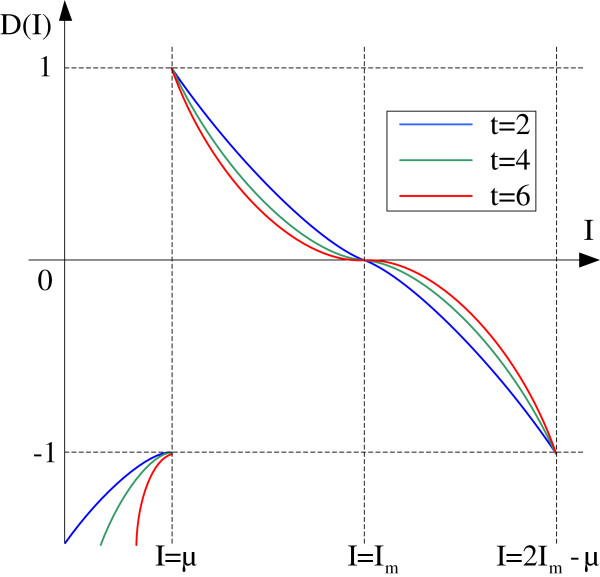
**The nonlinear piecewise data term in the new speed function.** Blue curve: the data term curve with t=2; Green curve: the data term curve with t=4; Red curve: the data term curve with t=6.

**Figure 7:**
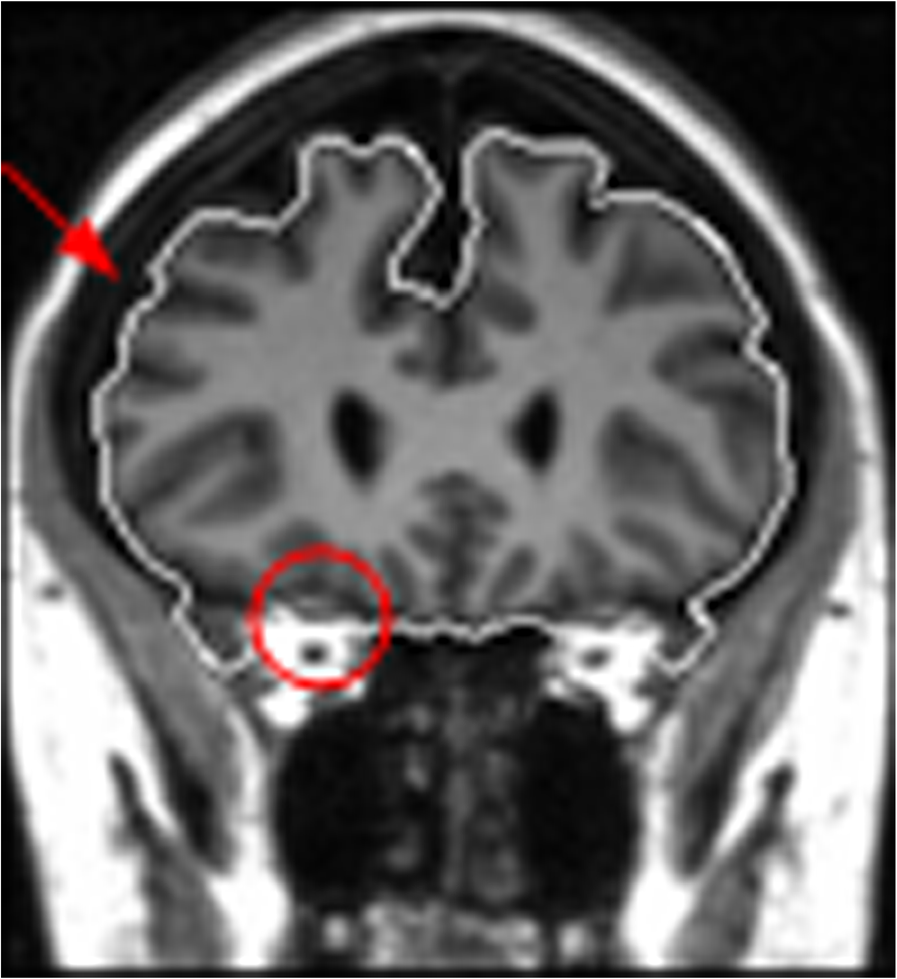
**A weak boundary between the brain tissues and eyeball tissues in the red ring.** The intensity of the region directed by the red arrow is lower than *μ* and the intensity of the region inside the red ring, with a weak boundary, is higher than Im.

In Figure 6, *μ* and *I*_*m*_ control the range of the grayscale values that can be considered to be located inside the brain tissues. Generally, the grayscale value outside the brain boundary but inside the ACN (the region indicated by the red arrow in Figure 7) is lower than μ. Hence, we set *D*(*I*) ≤ 0 to make sure the voxels with grayscale values in the interval [0 *μ*] were excluded by the contracting of the active contour. Observing that some of the surrounding tissues in the ACN, such as eyeball, have grayscale values bigger than *I*_*m*_ (the region enclosed by the red ring in Figure 7), if the contour leaks through a weak boundary in these areas, the contour can expand rapidly leading to a very poor result. To avoid this happening, we set *D*(*I*) ≤ 0 to make sure the voxels with grayscale values in the interval [*I*_*m*_ 1] were excluded by the contracting of the active contour. Whereas inside the interval [*μ I*_*m*_], *D*(*I*) > 0, the voxels in these areas are enclosed by the expanding of the active contour. The speed parameter is t. In Figure 6, t has the opposite effect on contour contraction and expansion in the interval [0 *I*_*m*_]. Increasing t can speed up contour contraction and slow down contour expansion, accordingly, decreasing t can slow down contour contraction and speed up contour expansion. When leakage occurs across a weak boundary, we can increase t to reduce the expansion rate and increase the contraction rate, thus the leakage can be corrected.

Compared with the normal hybrid level set model, the smoothing term in the speed function is defined by the regional value *I*_*r*_ not the image gradient. This is because the image gradient near a weak boundary is not big enough to stop the contour evolution, and the gradient between the white matter (WM) and the gray matter (GM) may be higher than that near the brain boundary because of strong noise and intensity inhomogeneity. If we used the image gradient in the smoothing term, the contour would easily leak though weak boundaries or stop at the intersection between the WM and GM. In Figure 7, the region indicated by the red arrow and the region inside the red ring at the edge of the brain boundary have large *I*_*r*_ values, which decreases the evolving speed of the active contour and avoids the contour leaking though weak boundaries.

## Results and discussion

### Data sets

To measure the extraction accuracy of our method, we used the following two data sets for performance evaluation:

(1). Data set 1: 18 normal T1-weighted MR image volumes with expert segmentations were obtained from the Internet Brain Segmentation Repository (IBSR) [21] developed by the Centre for Morphometric Analysis (CMA) at Massachusetts General Hospital. Each volume has around 128 coronal slices, with 256 × 256 pixels per slice and a slice thickness of 1.5 mm.

(2). Data set 2: 20 normal T1-weighted MR image volumes from IBSR, with 256 × 256 pixels per slice and a slice thickness of 3.1 mm.

Before performing evaluation, the ground truth of brain tissues must be defined first. Without a doubt the GM and WM are included in the ground truth and non-brain structures, such as the skull, dura, and eyes are excluded. However, there are different viewpoints on other structures and tissues, such as the amount of extra-cerebral and content such as cerebro-spinal fluid (CSF), blood vessels, and nerves to be included [11]. Some methods define the ground truth as the WM and GM only, while others include CSF, veins, and the optic chiasms. Considering that CSF in the T1-weighted MRI has low intensity and is easily separated from non-liquid structures, and that subsequent analyses may benefit from the inclusion of CSF as noted in [22], we followed the definition used in paper [11]:

Included in the ground truth

• All cerebral and cerebellar white matter

• All cerebral and cerebellar gray matter

• CSF in ventricles (lateral, 3rd and 4th) and the cerebellar cistern

• CSF in deep sulci and along the surface of the brain and brain stem

• The brainstem (pons, medulla)

Excluded from the ground truth

• Skull, skin, muscles, fat, eyes, dura mater, bone and bone marrow

• Exterior blood vessels – specifically the carotid arteries, the superior sagittal sinus and the transverse sinus

• Exterior nerves – specifically the optic chiasms

### Evaluation metrics

(1). Similarity coefficients. We used Jaccard similarity, defined as $\mathrm{JS}=\frac{\left|M\cap N\right|}{\left|M\cup N\right|}$, and Dice similarity, defined as $\mathrm{DS}=\frac{2\left|M\cap N\right|}{\left|M\right|+\left|N\right|}$, where M and N refer to the extraction result and the ground truth respectively.

(2). Segmentation error: False Positives Rate (FP_Rate) and False Negatives Rate (FN_Rate). we used $\mathrm{FP}\_\mathrm{Rate}=\frac{\left|M\right|-\left|M\cap N\right|}{\left|N\right|}$ and $\mathrm{FN}\_\mathrm{Rate}=\frac{\left|N\right|-\left|N\cap M\right|}{\left|N\right|}$.

(3). Hausdorff distance between M and N. The Hausdorff distance is the maximum distance of M to the nearest point in N, the definition of Hausdorff distance between M and N is

$$\mathrm{HD}\left(M,N\right)=\underset{a\in M}{\mathrm{MAX}}\left\{\underset{b\in N}{\mathrm{MIN}}\left(d\left(a,b\right)\right)\right\}$$

where a and b are the points in M and N respectively, and d(a, b) is the Euclidian distance between a and b.

### Comparison to other methods

A comparison to BET (The version in the MRIcro software), BSE (The version in the Brainsuite9.0 software), WAT, HWA, GCUT and ROBEX was performed on the two chosen data sets.

The software [23] (Additional file 1) developed for our research was programmed using matlab2009 and was tested on a computer with 3GB of RAM and an Intel i7-2600 CPU. The software took approximately 10 minutes to process each MRI volume. Using this software, only parameters *β* and *t* from equation (5) needed to be set by the user for each volume. In testing, we chose to use fixed parameters (*β*=1.2 and *t*=6) for both data sets (labeled “ACNM One” in Tables 1 and 2) then changed these two parameters for each MRI volume in data set 1 (labeled “ACNM Two” in Table 1). “ACNM Three” in Table 1 had the same optimal parameters as “ACNM Two”, but had different initial conditions.

**Table 1:** Comparison of ACNM with existing brain extraction approaches on data set 1: BSE in Brainsuite software, BET in MRIcro software, WAT, HWA, GCUT and ROBEX using IBSR data set 1 (18 × 1.5 mm scans)

**Method**	**DS mean (SD)[range]**	**JS mean (SD)[range]**	**FP_Rate(%) mean (SD)[range]**	**FN_Rate(%) mean (SD)[range]**	**HD mean (SD)[range]**
BSE	**0.94(0.04)**	**0.90(0.07)**	**7.8(6.2)**	**3.7(6.3)**	**16.5(6.7)**
	**[0.83-0.97]**	**[0.70-0.95]**	**[1.6-18.6]**	**[0.5-28.5]**	**[8-30]**
BET	**0.88(0.03)**	**0.78(0.04)**	**5.7(1.3)**	**17.5(4.9)**	**19.6(4.4)**
	**[0.86-0.96]**	**[0.72-0.93]**	**[4.4-8.9]**	**[1.0-24.9]**	**[7-29]**
WAT	**0.91(0.08)**	**0.85(0.11)**	**18.8(29.7)**	**2.45(1.79)**	**_**
	**[0.60-0.96]**	**[0.43-0.92]**	**[5.3-134.3]**	**[0.08-7.08]**	
HWA	**0.88(0.03)**	**0.79(0.04)**	**27.1(6.8)**	**0.015(0.02)**	**_**
	**[0.82-0.91]**	**[0.69-0.83])**	**[20.0-44.4])**	**[0-0.07]**	
GCUT	**0.91(0.02)**	**0.84(0.03)**	**19.3(4.0)**	**0.029(0.04)**	**_**
	**[0.87-0.93]**	**[0.78-0.87])**	**[14.8-28.6]**	**[0-0.15]**	
ROBEX	**0.96(0.08)**	**_**	**_**	**_**	**13.3(2.6)**
ACNM One	**0.95(0.02)**	**0.90(0.03)**	**7.04(3.68)**	**3.58(3.95)**	**12.4(4.5)**
	**[0.91-0.97]**	**[0.83-0.94]**	**[3.0-15.1]**	**[0.58-14.28]**	**[7-21]**
ACNM Two	**0.96(0.01)**	**0.92 (0.03)**	**5.28(2.06)**	**3.41(2.73)**	**10.5(3.0)**
	**[0.93-0.98]**	**[0.88-0.95]**	**[2.81-10.35]**	**[0.82-8.64]**	**[7-18]**
ACNM Three	**0.94(0.03)**	**0.90(0.05)**	**4.66(1.6)**	**6.15(5.86)**	**14.9(4.4)**
	**[0.85-0.98]**	**[0.74-0.95]**	**[1.21-8.11]**	**[1.88-11.17]**	**[7-24]**

“ACNM One” represents performing the ACNM with fixed parameters. “ACNM Two” represents performing the ACNM with varied parameters. “ACNM Three” represents initializing using a simple circle.

**Table 2:** Comparison of ACNM with existing brain extraction approaches on data set 2: BSE in Brainsuite software, BET in MRIcro software, WAT, HWA, GCUT and ROBEX using IBSR data set 2 (20 × 3.1 mm scans)

**Method**	**DS mean (SD)[range]**	**JS mean (SD)[range]**	**FP_Rate(%) mean (SD)[range]**	**FN_Rate(%) mean (SD)[range]**	**HD mean (SD)[range]**
BSE	**0.93(0.05)**	**0.88(0.08)**	**6.8(2.7)**	**6.3(9.5)**	**23.5(7.4)**
	**[0.75-0.96]**	**[0.60-0.93]**	**[4.2-12.5]**	**[1.1-36.5]**	**[10-30]**
BET	**0.85(0.08)**	**0.75(0.11)**	**22.9(8.0)**	**9.0(11.3)**	**25.4(7.0)**
	**[0.67-0.93]**	**[0.50-0.87]**	**[11.8-39.8]**	**[0.2-29.6]**	**[7-30]**
WAT	**0.76 (0.14)**	**0.64 (0.18)**	**18.4 (14.1)**	**24.5 (22.7)**	**_**
	**[0.47–0.92]**	**[0.31–0.86]**	**[5.2–61.2]**	**[0.1–62.7]**	
HWA	**0.78 (0.21)**	**0.68 (0.21)**	**131.2 (308.2)**	**1.9 (6.5)**	**_**
	**[0.16–0.88]**	**[0.09–0.78]**	**[19.4–1060.2]**	**[0.0–28.9]**	
GCUT	**0.85 (0.09)**	**0.75 (0.10)**	**38.3 (40.1)**	**0.01 (0.02)**	**_**
	**[0.49–0.90]**	**[0.33–0.81]**	**[23.1–207.5]**	**[0.0-0.06]**	
ROBEX	**0.96(0.08)**	**_**	**_**	**_**	**13.3(2.6)**
ACNM One	**0.94(0.02)**	**0.89(0.03)**	**9.14(4.6)**	**2.46(1.94)**	**12.6(5.6)**
	**[0.91-0.96]**	**[0.84-0.93]**	**[3.6-17.5]**	**[0.26-8.51]**	**[6-25]**

How the experimental results for the other methods were obtained is described as follows. For BET, we changed the fractional intensity parameter in the range [0.3 0.8] for each volume to obtain better performance. For BSE, we changed two parameters (diffusion constant and erosion size) when the default parameters led to bad extraction results for some volumes in data set 1 and 2. For WAT, HWA and GCUT, we used the experimental results with fixed parameters for each data set reported in [12]. For ROBEX, we used the experimental results with fixed parameters on the IBSR data sets reported in [13]. However, paper [13] didn’t illustrate which IBSR data set was used, so we used the same experimental results on data set 1 and data set 2.

In Tables 1 and 2, “ACNM One” with fixed parameters (*β=*1.2 and *t=*6) led to the best JS, DS and HD coefficients out of all of the methods listed. The DS using “ACNM One” was slightly lower than that using ROBEX, but this is not statistically significant. The FN_Rate and FP_Rate coefficients weren’t as good as those using other methods. Generally, the smaller the FN_Rate and FP_Rate are, the more accurate the extraction results. However, if the extraction results include all of the brain tissue and a lot of non-brain tissue, the FN_Rate equals 0. So an algorithm with a smaller FN_Rate and FP_Rate does not always produce a more accurate result. An accurate and robust algorithm must have a good trade-off between FN_Rate and FP_Rate. The FP_Rate and FN_Rate were lower than 10% using “ACNM One” and BSE, which indicates a favorable and acceptable trade-off between the FN_Rate and FP_Rate. The smallest HD coefficient produced using “ACNM One” suggests that it tends to preserve smaller non-brain structures in the vicinity of the brain surface, such as the dura, while the other methods preserve larger non-brain structures in the eye, neck and skull areas. Since “ACNM One” used the slice by slice initial method, slice thickness might affect the performance, and that may be why“ACNM One” did a better job of processing data set 1 with 1.5 mm thick slices than data set 2 with 3.1 mm thick slices, when the fixed parameters remained the same for both data sets. We changed the parameters (*β* and *t*) for each MRI volume in data set 1 to obtain more accurate results (“ACNM Two” in Table 1) and found that if the parameters were properly set, all of the evaluation metrics improved.

**Figure 8:**
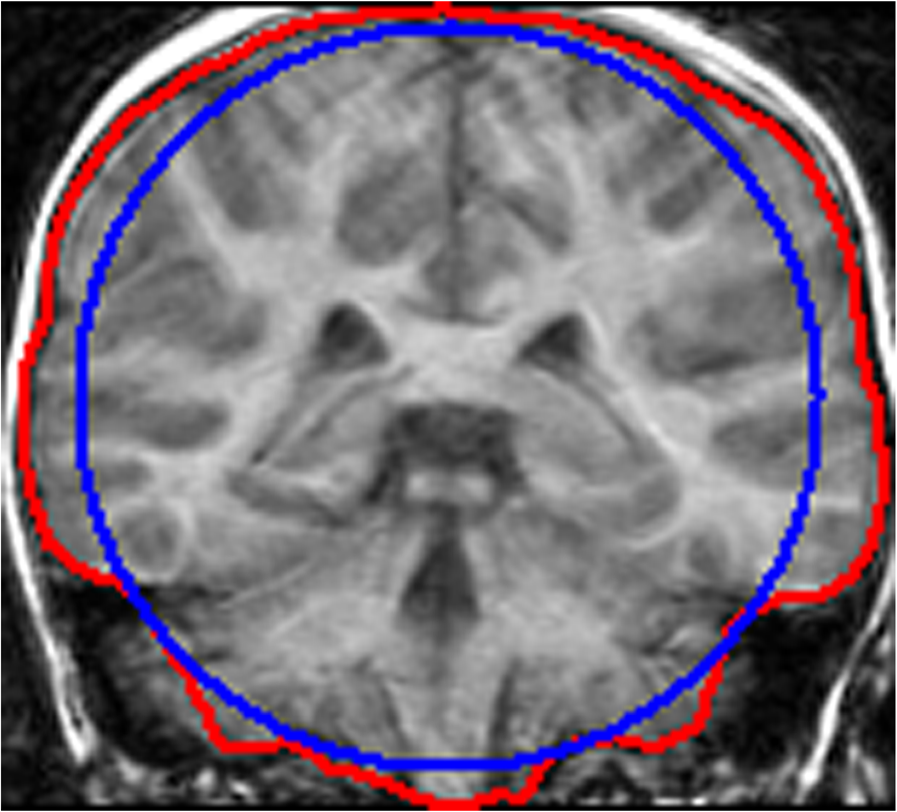
**Two different initial contours in the middle slice.** Red curve: the initial contour using the resultant contour of BET; Blue curve: the initial contour using a circle.

To prove how applying the slice by slice initial method to the middle slice impacts on the brain extraction result, two different initial methods were used in conjunction with our proposed ACNM method. In Table 1, “ACNM One” and “ACNM Two” initialized the contour in the middle slice using the resultant contour (the red curve in Figure 8) from BET, and then performed ACNM using the slice by slice method. “ACNM Three” initialized the contour in the middle slice with a circle contour (the blue curve in Figure 8) close to the resultant contour from BET, and then performed ACNM using the slice by slice method. The circle contour had the same centroid as the resultant contour from BET and the area of the circle was set as 0.83 times the area of the resultant contour from BET. Both “ACNM Two” and “ACNM Three” used the same optimal parameters for the each MRI volume in data set 1. As shown in Table 1, “ACNM Two” performed better than “ACNM Three”, which proves that initializing the contour in middle slice using the resultant contour from BET improves the brain extraction result.

**Figure 9:**
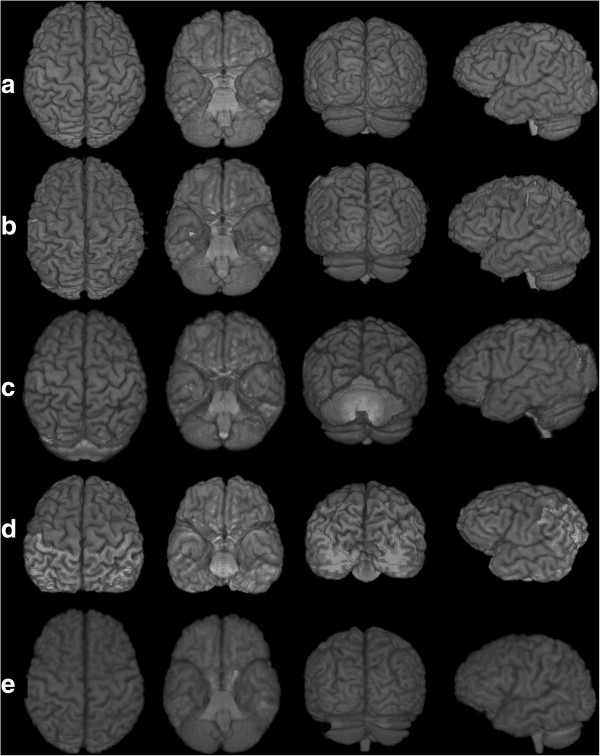
**Results of using the manual method, our method, GCUT, BET and BSE on the IBSR data.** (**a**) Manual method (**b**) Our method (**c**) GCUT (**d**) BET in MRIcro software (**e**) BSE in Brainsuite software.

Figure 9 shows the final results using our ACNM method, GCUT, BET and BSE on the eighth volume on data set 1 as well as the manual segmentation result. We used MIPAV [24] software to obtain 3D images from four different viewpoints. Compared to the manual result, the final results using our ACNM method have fewer boundary leakages on the brain surface than GCUT and clearer structures of the gyri, sulci and brainstem than BET and BSE, although there are some rough artifacts between the slices in our final 3D results due to the independent way each slice is processed. It is obvious that the results using GCUT showed some boundary leakages on the occipital pole, the results using BET missed the cerebellum, and the results using BSE were oversmoothed on the surface of the brain.

Compared to the other methods, the ACNM method used in our research led to fewer boundary leakages and more accurate contours close to the brain boundary. This was due to the more effective initialization method and more desirable hybrid level set model that we used in the ACN. As an automatic brain extraction method, our ACNM method is a considerably effective method with high accuracy and robustness.

## Conclusions

We proposed an accurate and robust brain extraction method using a hybrid level set based active contour neighborhood model (ACNM). The proposed ACNM method has the following advantages over existing brain extraction algorithms: Firstly, our method uses a slice by slice contour initial method based on BET for 3D MRI volumes. This initial approach can obtain a robust initial contour very close to the brain boundary, which ensures that the active contour evolves to the true brain boundary, thus an initial ACN including most of the true brain boundary can be defined. Secondly, the proposed method only evolves the contour using the new hybrid level set model in the ACN. Since the ACN is mainly in the neighborhood of true brain boundary, the resultant brain boundary can be obtained more accurately and robustly. Thirdly, the new hybrid level set model has a nonlinear speed function which can effectively eliminate boundary leakage. Thus, the proposed method can extract brain tissues with a higher accuracy and robustness when compared to other methods.

Although the proposed method has the above advantages, there are some disadvantages with the current implementation. Firstly, the computational cost is higher than the other methods so in future a GPU based accelerating technique will be applied to greatly reduce the computational time. Secondly, two parameters need to be set manually, and if these parameters are set incorrectly the extraction result may not be accurate. Estimating these parameters using a machine learning method is a future project. Thirdly, our method was implemented as a 2D algorithm and may have resulted in some rough artifacts between slices in the final 3D results. However, the experiments carried out in our research showed that the 2D algorithm achieved more accurate extraction results than some 3D algorithms by using the similarity between 2D cortical contours on adjacent slices, which matches the conclusion in [6]. How to eliminate these rough artifacts will be an important future project.

## Abbreviations

CN: Contour neighborhood; ACN: Active CN; ACNM: Active CN model; BET: Brain extraction tool; BSE: Brain surface extraction; WAT: Watershed algorithm; HWA: Hybrid watershed algorithm; GCUT: Graph cuts; ROBEX: Robust, learning-based brain extraction system; JS: Jaccard similarity; DS: Dice similarity; FP_Rate: False positives rate; FN_Rate: False negatives rate; HD: Hausdorff distance.

## Competing interests

The authors declare that they have no competing interests.

## Authors’ contributions

SJ worked on the algorithm design and development, and drafted the manuscript; WZ participated in the material preparation and experiment, YW revised the paper and ZC contributed discussions and suggestions throughout this project. All authors read and approved the final manuscript.

## Acknowledgements

This work was supported by the National Natural Science Foundation of China (Grant No. 61162023 and No.61163046), Education Department Technology Project of Jiangxi province in China (Grant No. GJJ10195) and National Natural Science Foundation of Jiangxi province in China (Grant No. 20114BAB211023).

## Supplementary Material

Additional file 1 Source code of ACNM. Click here for file

## References

[B1] Lemieux L Hagemann G Krakow K Woermann FG Fast, accurate, and reproducible automatic segmentation of the brain in T1-weighted volume MRI data Magn Reson Med 1999 42 127 135 10.1002/(SICI)1522-2594(199907)42:1<127::AID-MRM17>3.0.CO;2-O 10398958

[B2] Cox RW AFNI: Software for analysis and visualization of functional magnetic resonance neuroimages Comput Biomed Res 1996 29 162 73 10.1006/cbmr.1996.0014 8812068

[B3] Hahn HK Peitgen HO The skull stripping problem in MRI solved by a single 3D watershed transform Lect Notes Comput Sci 1935 2000 134 143

[B4] Smith SM Fast robust automated brain extraction Hum Brain Mapp 2002 17 143 155 10.1002/hbm.10062 12391568 PMC6871816

[B5] Shattuck DW Sandor-leahy SR Schaper KA Rottenberg DA Leahy RM Magnetic resonance image tissue classification using a partial volume model NeuroImage 2001 13 856 876 11304082 10.1006/nimg.2000.0730

[B6] Zhuang AH Valentino DJ Toga A Skull-stripping magnetic resonance brain images using a model-based level set NeuroImage 2006 32 79 92 10.1016/j.neuroimage.2006.03.019 16697666

[B7] Zhang HY Liu JF Zhu ZX Li HY An automated and simple method for brain MR image extraction Biomed Eng Online 2011 10 1 81 10.1186/1475-925X-10-81 21910906 PMC3180437

[B8] Ségonne F Dale AM Busa E Glessner M Salat D Hahn HK Fischl B A hybrid approach to the skull stripping problem in MRI NeuroImage 2004 22 1060 75 10.1016/j.neuroimage.2004.03.032 15219578

[B9] Huang A Abugharbieh R Ram R Traboulsee A RababWard and Fayez Gebali MRI brain extraction with combined expectation maximization and geodesic active contours Proceedings of the 6th IEEE International Symposium on Signal Processing and Information Technology: 27-30 August 2006 2007 Vancouver: Institute of Electrical and Electronics Engineers 107 111

[B10] Rex DE Shattuck DW Woods RP Narr KL Luders E Rehm K Stolzner SE Rottenberg DA Toga AW A meta-algorithm for brain extraction in MRI NeuroImage 2004 23 625 637 10.1016/j.neuroimage.2004.06.019 15488412

[B11] Eskildsen SF Coupé P Fonov V Manjón JV Leung KK Guizard N Wassef SN Østergaard LR Collins DL BEaST: Brain Extraction based on nonlocal Segmentation Technique NeuroImage 2012 59 3 2362 2373 10.1016/j.neuroimage.2011.09.012 21945694

[B12] Sadananthan S Zheng W Chee M Zagorodnov V Skull stripping using graph cuts NeuroImage 2010 49 1 225 239 10.1016/j.neuroimage.2009.08.050 19732839

[B13] Iglesias JE Liu CY Thompson PM Robust brain extraction across datasets and comparison with publicly available methods IEEE Trans Med Imaging 2011 30 9 1617 1634 21880566 10.1109/TMI.2011.2138152

[B14] Xu N Bansal R Ahuja N Danielle M Object segmentation using graph cuts based active contours Proceedings of IEEE International Conference on Computer Vision and Pattern Recognition: 18-20 June 2003, Volume 2 2003 Madison: Wisconsin 46 53

[B15] Jiang SF Yang SY Chen Z Chen WF Shi RY Automatic extraction of brain from cerebral MR image based on improved BET method 2nd international conference on biomedical engineering and informatics: 17-19 October 2009 2009 Tianjin, China: Institute of Electrical and Electronics Engineers 132 135

[B16] Juan O Keriven R Postelnicu G Stochastic motion and the level set method in computer vision: Stochastic active contours Int J Comput Vis 2006 69 1 7 25 10.1007/s11263-006-6849-5

[B17] Xie X Mirmehdi M MAC: Magnetostatic active contour model IEEE Trans Pattern Anal Mach Intell 2008 30 4 632 645 18276969 10.1109/TPAMI.2007.70737

[B18] Wang T Cheng I Basu A Fluid Vector Flow and Applications in Brain Tumor Segmentation IEEE Trans. on BME 2009 56 3 781 789 10.1109/TBME.2009.2012423 19174335

[B19] Zhang Y Matuszewski BJ Shark LK Moore CJ Chris M, Georges G, Urska C, Marjan T, Feng D Medical image segmentation using new hybrid level-set method Proceedings of the 5th IEEE International Conference on Biomedical Visualisation: 9-10 July 2008 2008 London: Institute of Electrical and Electronics Engineers 71 76

[B20] Lefohn AE Kniss JM Hansen CD Whitaker RT A streaming narrow-band algorithm: interactive computation and visualization of level sets IEEE Trans Vis Comput Graph 2004 10 4 422 433 10.1109/TVCG.2004.2 18579970

[B21] The IBSR Databasehttp://www.cma.mgh.harvard.edu/ibsr/

[B22] Carass A Cuzzocreo J Wheeler MB Bazin P-L Resnick SM Prince JL Simple paradigm for extra-cerebral tissue removal: Algorithm and analysis NeuroImage 2011 56 4 1982 1992 10.1016/j.neuroimage.2011.03.045 21458576 PMC3105165

[B23] The software developed for our researchhttp://www.freedrive.com/file/1706893

[B24] The MIPAV softwarehttp://mipav.cit.nih.gov

